# Nanostructured Lipid Carriers Loaded with Donepezil for Nose-to-Brain Targeting

**DOI:** 10.3390/pharmaceutics18050541

**Published:** 2026-04-28

**Authors:** Isabelly Fernanda Ferraz de Souza, Rodrigo Vicentino Placido, Maria Júlia Placido, Letícia Carvalho Rocha, Rudy Bonfilio, Vanessa Bergamin Boralli, André Luís Morais Ruela, Gislaine Ribeiro Pereira

**Affiliations:** 1 Faculty of Pharmaceutical Sciences, Federal University of Alfenas, Alfenas 37130-001, MG, Brazil; isabelly.souza@sou.unifal-mg.edu.br (I.F.F.d.S.); rodrigo.placido@sou.unifal-mg.edu.br (R.V.P.); maria.placido@sou.unifal-mg.edu.br (M.J.P.); leticia.rocha@sou.unifal-mg.edu.br (L.C.R.); rudy.bonfilio@unifal-mg.edu.br (R.B.); vanessa.marques@unifal-mg.edu.br (V.B.B.); 2 Pharmacy School, Federal University of Ouro Preto, Ouro Preto 35400-000, MG, Brazil; andre.ruela@ufop.edu.br

**Keywords:** Alzheimer’s disease, donepezil, nanostructured lipid carriers, nose to brain, pharmacokinetics, intranasal administration

## Abstract

**Background/Objectives:** The oral administration of donepezil has been shown to have common side effects due to systemic drug delivery, with fluctuations in blood and brain donepezil concentrations. Therefore, we obtained nanostructured lipid carriers loaded with donepezil (donepezil–NLC) for nose-to-brain targeting. **Methods:** The obtained NLCs were characterized by measurements of particle size, the polydispersity index, zeta potential, encapsulation efficiency, atomic force microscopy, Differential Scanning Calorimetry, Fourier transform infrared spectroscopy, X-ray diffraction, and in vitro release studies. Plasma and brain pharmacokinetic studies in Wistar rats were carried out to determine brain targeting. **Results:** Donepezil–NLC showed low polydispersity and nanometric size, high zeta potential, and high drug entrapment efficiency. Microscopy images showed spherical particles with regular surfaces. Thermal analysis, X-ray diffraction, and FTIR-ATR suggested the formation of an amorphous lipid matrix and the incorporation of donepezil molecularly dispersed within the lipid matrix. In vitro drug release studies demonstrated a biphasic drug release pattern with an initial burst followed by sustained release, with results better fitted to the Korsmeyer–Peppas model (*n*-value > 0.5). Following the nasal administration of donepezil–NLC, brain pharmacokinetic studies in Wistar rats demonstrated a significant improvement in bioavailability. Compared to the intravenous injection of donepezil, the AUC^0–ꝏ^ value was 10.5-fold higher. Drug targeting efficiency and direct transport percentage showed extremely higher values, suggesting nose-to-brain targeting after donepezil–NLC intranasal administration. **Conclusions:** Donepezil–NLC has proven to be an efficient drug delivery system for the nose to the brain, which may reduce systemic toxicity and improve Alzheimer’s therapy with low doses of donepezil and fewer adverse effects.

## 1. Introduction

Dementia is proving to be the major global public health crisis of the 21st century, affecting 50 million patients worldwide in 2010 and possibly reaching 152 million in 2050. Alzheimer’s disease is the most common cause of dementia, is incurable, and has a multifactorial nature [1,2,3,4,5,6].

Donepezil, an acetylcholinesterase inhibitor, is the most commonly used drug for treating Alzheimer’s disease. It is administered orally via coated tablets (5 or 10 mg). However, commercially available oral donepezil tablets cause gastrointestinal side effects, such as diarrhea, bradycardia, vomiting, insomnia, nausea, and anorexia. Furthermore, donepezil undergoes first-pass hepatic metabolism following oral administration, and a large drug dose must be administered, leading to considerable fluctuations in drug plasma concentrations. Therefore, novel drug delivery systems for donepezil administration via alternative routes are required to overcome the limitations of the oral route [7,8,9,10].

The nasal administration of nanostructured lipid carriers (NLCs) is reportedly effective, particularly in treating neurological disorders [11,12,13,14,15,16,17]. The nasal route is noninvasive, easily accessible, and painless and avoids first-pass hepatic metabolism. Drugs targeting the brain may be reached by the nasal administration of NLCs, overcoming the low permeability of the blood–brain barrier (BBB) and increasing the brain concentrations of the drug. Additionally, the lipid nature of nano-sized particles improves drug permeability through biological membranes [9,13,16,18,19,20,21,22,23,24].

Currently, the advantages of the nasal administration of NLCs loaded with drugs have been primarily studied for the treatment of pathologies in the central nervous system, such as migraine, insomnia, anxiety, brain tumors, meningoencephalitis, neuropathic pain, Parkinson’s disease, schizophrenia, and dementia, such as Alzheimer’s disease [20,25,26,27,28,29,30,31,32].

One of the most important advantages of employing this method is the significant improvement in brain bioavailability, as evidenced by an increase in C_max_ and AUC. In addition to imaging investigations, nose-to-brain targeting has been demonstrated by estimating drug targeting efficiency (DTE) and direct transport from the nose to the brain (DTP) [14,17,25,29,30,33,34,35].

Preclinical studies have demonstrated that the intranasal administration of NLCs improves various brain disorders. Seizure onset time in epilepsy increased while seizure length decreased, the period of immobility was reduced, the duration of struggle increased in depression, and the nociceptive consequences of neuropathic pain were amplified and lasted longer. In dementia, there was an improvement in and regression of cognitive deficiency, in addition to a substantial reduction in oxidative stress and inflammatory cytokines [27,28,36,37]. In a recent study, the analgesic efficacy of donepezil–NLC (presented in this work) intranasally administered for neuropathic pain was evaluated, focusing on the involvement of cholinergic receptors [32].

The nasal administration of NLCs may significantly improve the brain bioavailability of donepezil, enabling the administration of lower doses of the drug, resulting in fewer adverse effects and more effective treatment for Alzheimer’s disease [8,23]. Given the disadvantages of the oral administration of donepezil, this study proposes obtaining nanostructured lipid carriers loaded with donepezil (donepezil–NLC) to perform the targeted delivery of the drug to the brain through nasal administration.

## 2. Materials and Methods

### 2.1. Materials

Donepezil hydrochloride in raw material was kindly gifted by Cristália Produtos Químicos and Farmacêuticos (Itapira, Brazil). Oleic acid (≥99%), stearic acid (≥95%), and Pluronic F68^®^ were purchased from Sigma-Aldrich (St. Louis, MO, USA). Tween 80^®^ was purchased from Vetec (Rio de Janeiro, Brazil). All other reagents used were of analytical grade.

### 2.2. Methods

#### 2.2.1. Preparation of NLC

Donepezil–NLC was prepared using the hot microemulsion technique [38]. A mixture of stearic acid (0.30 g), oleic acid (0.10 g), Tween 80^®^ (0.10 g), and Pluronic F68^®^ (0.10 g) was heated to 70 °C, and 0.10 g of donepezil (to obtain 5 mg. mL^−1^ in the NLC dispersion) was added to the molten material. Next, 400 μL of distilled water was added under magnetic stirring (500 rpm) for 2 min to obtain a hot microemulsion. The hot microemulsion was then dripped into 20 mL of isotonic phosphate buffer (10 mmol, pH 7.5, 0.9% *w*/*v* sodium chloride) at 2–4 °C under vigorous stirring (16,000 rpm for 15 min, T25 Ultra-Turrax^®^, IKA Werke GmbH & Co, Staufen im Breisgau, Germany) to generate a donepezil–NLC dispersion. Unloaded NLCs were used as the controls. After preparation, the pH and osmolarity of the formulations were evaluated using a pH meter (MPA 210 PH METER, MS TECNOPON^®^, Piracidaba, Brazil) and an osmometer (Osmometer Model 3250, Advanced Instruments, Norwood, MA, USA), respectively. For the DSC, X-ray diffraction, and FTIR-ATR analyses, the NLC dispersions were previously lyophilized at −49 °C under a pressure of 0.024 mBar, and mannitol (5% *w*/*w*) was used as a cryoprotectant in the freeze-drying process.

#### 2.2.2. NLC Characterization

##### Hydrodynamic Diameter, Polydispersity Index, and Zeta Potential

The average hydrodynamic diameter and polydispersity index (PdI) of the NLC dispersions were determined using dynamic light scattering (DLS). The zeta potential was determined by measuring electrophoretic mobility. Both measurements were performed using a Zetasizer Nano ZS (Malvern Instruments Ltd., Malvern, UK). All samples were diluted (1:100) in purified water, and measurements were performed at 25 °C. The conductivity of the samples was approximately 50 µS cm^−1^ (20 °C).

##### Drug Entrapment Efficiency (%) and Drug Loading (%)

Drug entrapment efficiency (EE) and drug loading (DL) refer to the amount of drug encapsulated by nanocarriers, and these parameters are expressed as percentages. EE is the amount of drug encapsulated compared to the total amount of drug initially added, while DL refers to the amount of drug encapsulated compared to the amount of lipids. EE and DL were indirectly determined by the ultrafiltration/centrifugation method using Amicon^®^ ultrafiltration devices (Merck Millipore, Burlington, MA, USA; 50 kD cut-off). One milliliter of the donepezil–NLC dispersion was centrifuged at 3400 rpm for 30 min to separate the free drug. The donepezil–NLC retained on the filter was washed three times with buffer to remove any free drug. The amount of donepezil in the filtered pool (free drug) was quantified by HPLC. The amount of donepezil in the donepezil–NLC dispersion (total drug) was obtained by diluting the donepezil–NLC dispersion in methanol, followed by HPLC. EE (%) and DL (%) were calculated using the following equations:

EE (%) = (total drug-free drug)/(total drug) × 100

DL (%) = (total drug-free drug)/(weight of lipids) × 100

##### Atomic Force Microscopy

Nanoparticles were visualized by atomic force microscopy (AFM) using a Park NX10 microscope (Park Systems, Suwon, South Korea). Aliquots (10 μL) of donepezil–NLC and unloaded NLC dispersions were deposited on mica plates and dried at room temperature. Images were obtained in non-contact operating mode with a silicon probe (model NSC15) and a cantilever resonance frequency of 277.39 kHz.

##### Differential Scanning Calorimetry

Differential Scanning Calorimetry (DSC) measurements were performed using a DSC Syrius 3500 instrument (Netzsch-Gerätebau GmbH, Selb, Germany). Samples (3 mg) of freeze-dried NLC formulations (donepezil–NLC and unloaded NLCs), stearic acid (solid lipid), donepezil hydrochloride, and mannitol (cryoprotectant used in the freeze-drying process) were placed in sealed aluminum pans. DSC curves were recorded from 25 °C to 300 °C at a heating rate of 10 °C.min^−1^ under a nitrogen atmosphere (50 mL.min^−1^).

##### Powder X-Ray Diffraction

Powder X-ray diffraction (PXRD) analyses were performed on freeze-dried NLC formulations (donepezil–NLC and unloaded NLCs), stearic acid, donepezil hydrochloride, and mannitol. A stearic acid: donepezil hydrochloride: mannitol mixture at a ratio of 3:1:10 (*w*/*w*/*w*) was also analyzed. Diffraction patterns were obtained at room temperature using an Ultima IV model X-ray diffractometer (Rigaku^®^, Akishima, Japan) with θ–2θ geometry under the following conditions: Cu Kα radiation (λ = 1.5418 Å) generated in a sealed tube and scans in the 2θ range of 3–35° with a 0.02° optical step, a current of 30 mA, a voltage of 40 kV, and a scan rate of 1° 2θ min^−1^.

##### FTIR-ATR Spectroscopy

The FTIR-ATR spectra of freeze-dried NLC formulations (donepezil–NLC and unloaded NLCs), stearic acid, donepezil hydrochloride, mannitol, and the stearic acid: donepezil hydrochloride: mannitol mixture at a ratio of 3:1:10 (*w*/*w*/*w*) were obtained using an Affinity-1 Fourier transform infrared spectrophotometer (Shimadzu, Tokyo, Japan) coupled to a Pike Miracle attenuated total reflectance sampling accessory with ZnSe waveguides (Pike Technologies, Madison, WI, USA). The spectra were recorded at room temperature using 32 scans at a resolution of 4 cm^−1^ in the range of 4000–600 cm^−1^.

##### Rheological Analysis

The rheological behavior of the donepezil–NLC dispersion was analyzed at 25 °C using an R/S-controlled stress rheometer (Brookfield Engineering Laboratories, Middleboro, MA, USA) and Rheocalc T 1.2.19 B software. Rheological measurements were performed using a CP 42 spindle with a shear gradient speed of 4–25 rpm. The relationship between the shear stress and shear rate of the formulation was evaluated using the following power law equation:

τ = 〖K γ〗^^η^

where τ is the shear stress (D.cm^−2^), K is the flow consistency index (centipoise, cP), γ is the shear rate (s − 1), and η is the flow index. Data were adjusted using the coefficient of determination (r2). For a Newtonian fluid, η = 1, and for a pseudoplastic fluid, η < 1.

#### 2.2.3. In Vitro Drug Release Studies

In vitro drug release studies on donepezil–NLC were performed using Franz-type diffusion cells (Hanson Research Corporation, EUA) and a regenerated cellulose acetate dialysis membrane (Spectra/Por^®^, 12–14 KDa cut-off, Spectrum Laboratories, Inc., Rancho Dominguez, CA, USA). Phosphate buffer (20 mM, pH 5), stirred at 300 rpm and thermostated at 37 °C, was used as the release medium to ensure sink conditions. At predetermined time intervals (0.25, 0.5, 0.75, 1, 1.5, 2, 2.5, 3, 3.5, 4, 5, and 6 h), aliquots were drawn from the receptor compartment, and the concentration of released donepezil was assayed by HPLC using a validated method described previously [39].

#### 2.2.4. Preliminary Stability Studies

To evaluate the physical stability of donepezil–NLC dispersions, freshly prepared samples were stored in a refrigerator (4 ± 0.5 °C), and the hydrodynamic diameter, PdI, and zeta potential were analyzed for 6 months. EE (%) was also determined after six months of storage.

#### 2.2.5. Plasma and Brain Pharmacokinetic Studies

This study was approved by the Animal Use Ethics Committee of the Federal University of Alfenas (protocol number 14/2020). The experiments were carried out on healthy adult male Wistar rats weighing between 200 g and 250 g, acquired from the university’s facilities. The animals were housed in polypropylene boxes, five in each box, at 20 ± 2 °C and 50% ± 5% relative humidity, with a 12/12 h light/dark reversed cycle. The animals were fed a regular rodent diet and provided unlimited access to tap water. Rats were divided into two groups of 42 rats each. The first group received donepezil solution at a dosage of 3 mg kg^−1^ intravenously. For intravenous administration, donepezil was dissolved in isotonic saline solution and administered via tail vein injection as a bolus dose. The second group received the donepezil–NLC dispersion (3 mg.kg^−1^) intranasally. For nasal administration, the animals were held by the skin in the cervical region and kept in a dorsal position at an angle of 45 º. Approximately 35 μL of formulation was pipetted to each nostril of the animal, and after 3 s, the procedure was repeated. In this study, six rats were used in each group at each time point. The rats were sedated after treatment with an intraperitoneal injection of thiopental (100 mg kg^−1^) and euthanized by decapitation at predefined periods (0.5, 1, 1.5, 3, 4, 6, and 8 h). Blood was collected immediately and placed in tubes containing heparin. Blood samples were centrifuged for 10 min at 4 °C and 2500 rpm to produce plasma supernatants, which were preserved at −80 °C. Brain samples were promptly collected, weighed, and frozen at −80 °C until processing. Plasma and brain samples were prepared using a previously established method and analyzed using UHPLC/MS-MS [40].

##### Calculation of Pharmacokinetic Parameters

At each time point, data on plasma and brain concentrations (n = 6) were used to estimate pharmacokinetic parameters using non-compartmental pharmacokinetic analysis performed with the PKSolver v. 2.0 for Excel (Microsoft^®^) add-in. The maximum peak concentration (C_max_), time to achieve C_max_ (T_max_), area under the drug concentration–time curve from time zero to infinity (AUC_0–∞_), half-life (t_1/2_), and clearance (Cl) were estimated as pharmacokinetic parameters.

##### Calculation of Drug Targeting Indices

Drug targeting efficiency (DTE) and direct transport percentage from the nose to the brain (DTP) were employed to select the drugs targeting the brain following intranasal administration [12,41,42,43]. DTE measures drug accumulation in the brain following intranasal administration (IN) compared to intravenous administration (IV). DTE was calculated using the following equation:

DTE% = (AUC_brain_, IN)/(AUC_blood_, IN) × 100

(AUC_brain,_ IV)/(AUC_blood_, IV)

DTE% values can range from 0 to ∞, and efficient brain targeting after IN administration compared to IV administration is suggested by results above 100%. The DTP parameter indicates the percentage of the drug targeting the brain via a direct route through the olfactory and trigeminal pathways and is determined using the following equation:

DTP% = AUC_brain_, IN-F × 100

AUC_brain_, IN

Here,

F = AUC_brain_, IV × AUC_blood_, IV

AUC_blood_, IV

DTP% values may range from −∞ to 100, and positive results up to 100% suggest excellent direct nasal drug transport to the brain, while lower results, the closest to 0, indicate that the drug reaches the brain uniquely through systemic circulation, similarly to IV administration.

#### 2.2.6. Statistical Analysis

The results from in vitro studies were expressed as the mean ± standard deviation, whereas pharmacokinetic data were expressed as the median and confidence intervals due to their non-normal distribution. Comparisons between groups were performed using the nonparametric Mann–Whitney U test. A *p*-value ≤ 0.05 was considered statistically significant (InStat 5.01 GraphPad Inc., San Diego, CA, USA).

## 3. Results

### 3.1. Preparation of NLCs

In this study, we obtained NLCs for the nasal administration of donepezil for nose-to-brain targeting. NLC dispersions were prepared with a total lipid content of 2.0% (1.5% stearic acid and 0.5% oleic acid) and stabilized using 1.0% surfactant (0.5% Tween 80^®^ and 0.5% Pluronic F68^®^).

The nasal liquid formulations must have a pH value within the range of the nasal mucosa (5.5–6.5) and an osmotic pressure of approximately 280 mOsm.kg^−1^ [44,45]. Therefore, donepezil–NLC was prepared in phosphate buffer (10 mmol, pH 7.5) with sodium chloride (0.9% *w*/*v*), resulting in a formulation with pH = 6.3 and 280 mOsm.kg^−1^, compatible with the nasal mucosa.

### 3.2. NLC Characterization

#### 3.2.1. Particle Size, PdI, Zeta Potential, EE, and DL

The NLCs were characterized by their average hydrodynamic diameter, PdI, zeta potential, EE, and DL. The results presented in Table 1 demonstrate a homogeneous size in nanometers. The zeta potential values obtained were greater than |30| mV, which contributed to the electrostatic stabilization of the colloidal dispersion. Donepezil–NLC showed high EE and DL values, which were attributed to the high lipophilicity of the drug.

#### 3.2.2. Atomic Force Microscopy

**Figure 1:**
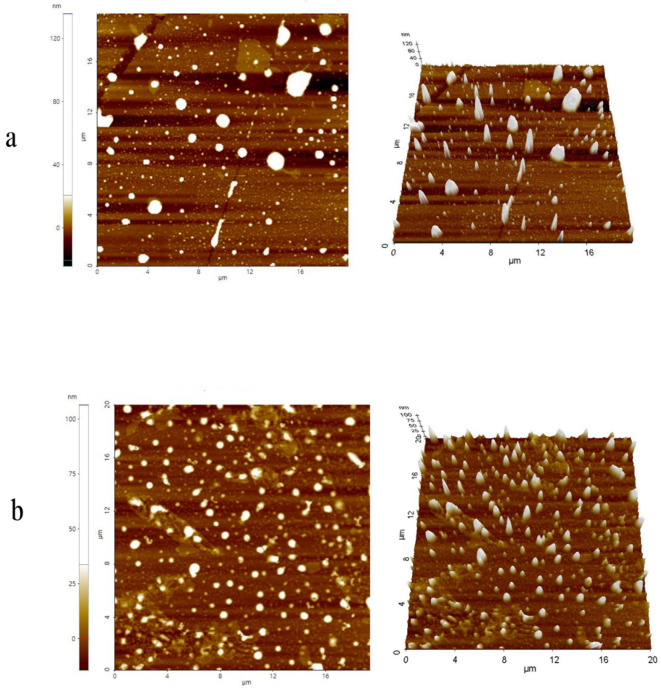
AFM images of unloaded NLCs (**a**) and donepezil–NLC (**b**): 2D surface topography and 3D particle distribution. The images were obtained using the true non-contact mode, with an n-type silicon probe (model NSC15) and a cantilever resonance frequency of 270.45 kHz.

The surfaces of donepezil–NLC and unloaded NLCs were examined using AFM, and the findings are presented in Figure 1. The NLCs showed a spherical shape and a regular surface. The average particle size measurement by AFM showed unloaded NLCs at 103.42 ± 44.98 nm and donepezil–NLC at 89.53 ± 26.82 nm.

#### 3.2.3. DSC, PXRD, and FTIR-FTIR Spectroscopy

**Figure 2:**
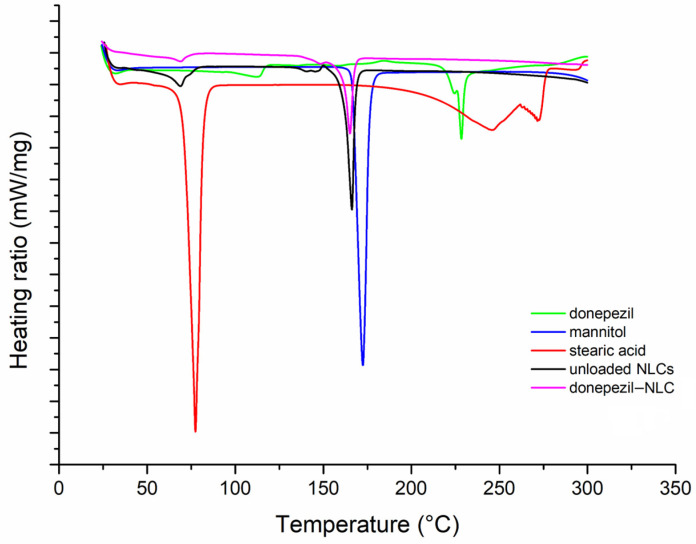
The DSC curves of mannitol, stearic acid, donepezil, donepezil–NLC, and unloaded NLCs, recorded from 25 °C to 300 °C at a heating rate of 10 °C/min under a nitrogen atmosphere (50 mL/min).

DSC and PXRD studies were performed to investigate the solid nature and crystallinity of the NLCs, as well as the drug–lipid interactions in the formulation. Figure 2 shows the DSC curves for donepezil, stearic acid (solid lipid), mannitol (cryoprotectant), and freeze-dried NLCs (donepezil–NLCs and unloaded NLCs). The DSC curves of donepezil, stearic acid, and mannitol show endothermic events at 228.7 °C, 77.7 °C, and 172.4 °C, respectively, corresponding to the melting point of these compounds, which demonstrated their crystalline nature. Stearic acid also decomposes at 200 °C.

The DSC curve of unloaded NLCs shows endothermic events at 68.10 °C and 165.08 °C. The first event can be attributed to stearic acid fusion, which was dislocated from 77.7 °C in crystalline stearic acid, which may be indicative of the reduction in crystallinity attributed to the formation of the NLC matrix, which is composed of a mixture of solid and liquid lipids. The second event at 165.08 °C may be attributed to the mannitol used as a cryoprotectant in the freeze-drying process. The DSC curve of donepezil–NLC showed endothermic events at 68.4 °C and 165.92 °C. The first event was similar to that observed in the unloaded NLCs, corresponding to stearic acid fusion in the NLC matrix. The second event was mannitol fusion, as observed in the unloaded NLCs. Additionally, an endothermic event related to the melting point of donepezil was not observed in the DSC curves of donepezil–NLC, suggesting that the drug is in an amorphous state or molecularly dispersed in the lipid matrix [46,47].

**Figure 3:**
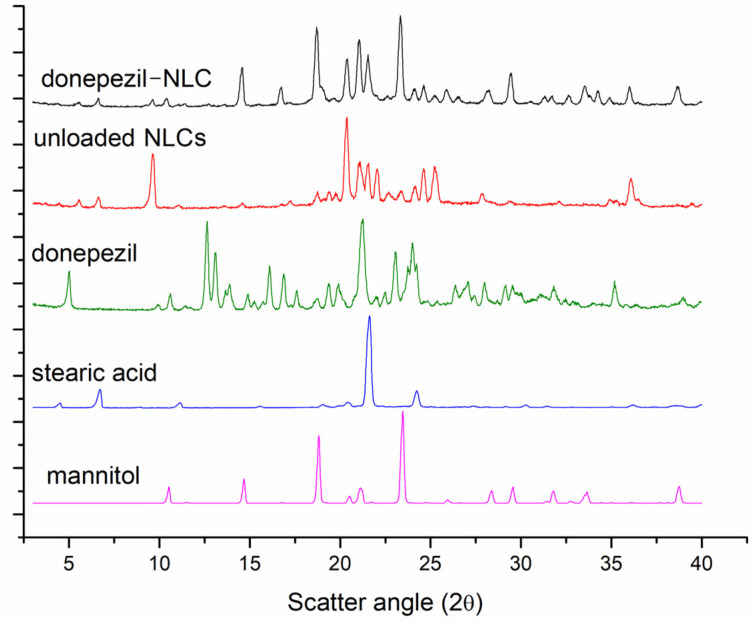
Powder X-ray diffraction patterns of donepezil–NLC, unloaded NLCs, donepezil, stearic acid, and mannitol.

The X-ray diffraction patterns of mannitol, stearic acid, donepezil, donepezil–NLC, and unloaded NLCs are shown in Figure 3. Intense sharp peaks for donepezil (5.0°, 9.9°, 10.5°, 11.5°, 12.5°, 13.8°, 14.6°, 16.1°, 17.0°, 17.6°, 18.8°, 19.0°, 21.2°, 23.4°, 24.0°, 26.0°, 27.0°, 28.4°, 29.6°, 31.8°, 35.2°, and 38.9°), stearic acid (4.5°, 6.7°, 8.9°, 11.1°, 15.5°, 19.0°, 20.5°, 21.6°, 24.3°, 27.5°, 29.2°, 30.2°, 31.5°, 32.9°, 35.1°, 36.2°, and 38.5°), and mannitol (10.5°, 11.5°, 14.6°, 16.8°, 18.9°, 21.1°, 23.4°, 24.7°, 26.0°, 28.3°, 29.5°, 31.8°, 32.8°, 33.7°, and 38.8°) demonstrate the crystalline nature of these components. Furthermore, the unloaded NLCs and donepezil–NLC demonstrated similarities. The unloaded NLCs showed intense sharp peaks for stearic acid (5.5°, 6.6°, 9.6°, 11.1°, 19.4, 18.7°, 20.4°, 21.5°, 24.2°, 27.8°, 36.1°) and mannitol (14.6°, 17.2°, 18.7°, 21.2°, and 24.5°) and unprecedented peaks (13.6°, 19.8°, 22.2°, 22.6°, and 25.3°). Donepezil–NLC exhibited peaks for stearic acid (5.6°, 6.6°, 9.6°, 20.4°, 24.2°, 26.6°, and 36.1°) and mannitol (14.6°, 16.8°, 24.6°, 32.6°, and 33.6°) and unprecedented peaks (25.2°, 31.4°, and 34.3°). As seen from the results above, some peaks were dislocated, and new peaks were observed for donepezil–NLC and unloaded NLCs, suggesting the formation of a new crystalline structure.

**Figure 4:**
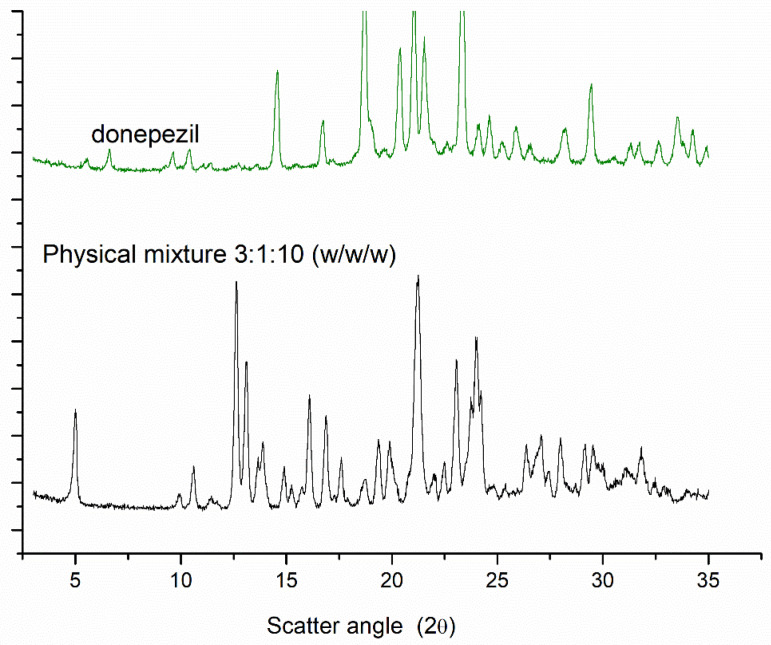
Powder X-ray diffraction patterns of donepezil and physical mixture of stearic acid, donepezil, and mannitol in 3:1:10 (*w*/*w*/*w*) proportions.

In addition, the exclusive peaks of the donepezil raw material (12.5°, 13.8°, and 17.6°) were not observed in the diffraction pattern of donepezil–NLC. These data may be attributed to the incorporation of donepezil into the crystal lattice of lipids; however, they can also indicate a low concentration of donepezil in the lipid matrix. A physical mixture of stearic acid, donepezil, and mannitol was analyzed in the ratio 3:1:10 *w*/*w*/*w* (ratio equal to lyophilized donepezil–NLC). A comparison of the PXRD results is shown in Figure 4. The peaks of donepezil raw material (12.5°, 13.8°, and 17.6°) were observed in the diffraction pattern of the physical mixtures, demonstrating the ability of this technique in analyzing the drug in the proportion of NLC loaded with donepezil.

**Figure 5:**
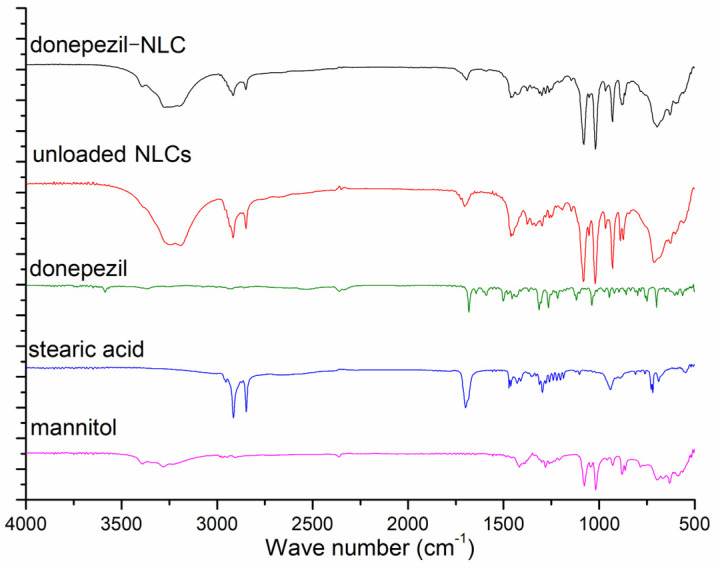
ATR-FTIR spectra of mannitol, stearic acid, donepezil, donepezil–NLC, and unloaded NLCs.

**Figure 6:**
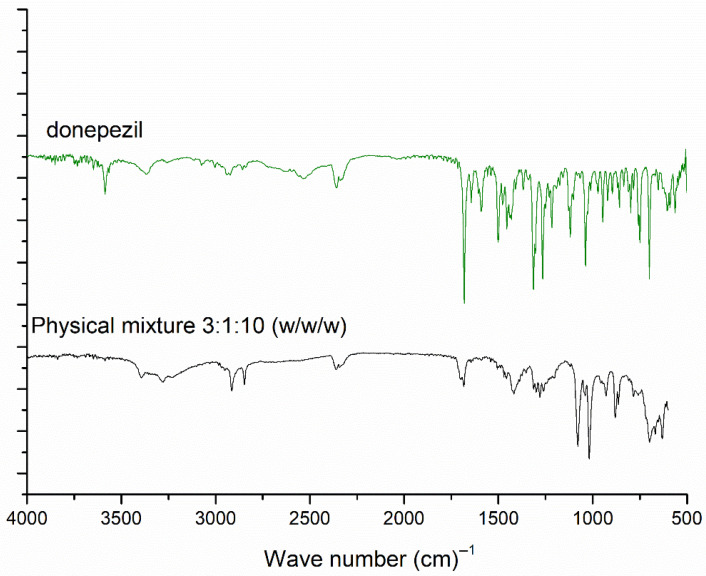
ATR-FTIR spectra of donepezil–NLC and physical mixtures of stearic acid, donepezil, and mannitol in proportions 3:1:10 (*w*/*w*/*w*), 3:2:10 (*w*/*w*/*w*), and 3:3:10 (*w*/*w*/*w*).

FTIR analyses were performed to investigate the physicochemical interactions between the drug and lipids in the NLCs. The results of the FTIR analysis are shown in Figure 5. Upon comparing the FTIR spectra of the unloaded NLCs and donepezil–NLC, we observed that both bands were attributed to mannitol (3038–3469 cm^−1^; 1462 cm^−1^; 1001–1104 cm^−1^) and stearic acid (2961–2836 cm^−1^; 1694 cm^−1^). Additionally, the bands in the spectrum typically associated with donepezil (3436–3320 cm^−1^; 3603–3554 cm^−1^; 1599–1580 cm^−1^) were absent in the spectra of the unloaded NLCs and donepezil–NLC, suggesting that the drug was incorporated into the lipid matrix of the NLCs. To evaluate the sensitivity of the technique for detecting the drug in donepezil–NLC samples, a physical mixture of stearic acid, donepezil, and mannitol was analyzed at the ratio 3:1:10 *w*/*w*/*w* (ratio equal to lyophilized donepezil–NLC) (Figure 6). Similarly to the PXRD analysis, the FTIR analysis of the physical mixture showed characteristic drug bands, demonstrating that under the conditions employed, this technique could identify the drug in the presence of the other components of the sample.

#### 3.2.4. Rheological Analysis

**Figure 7:**
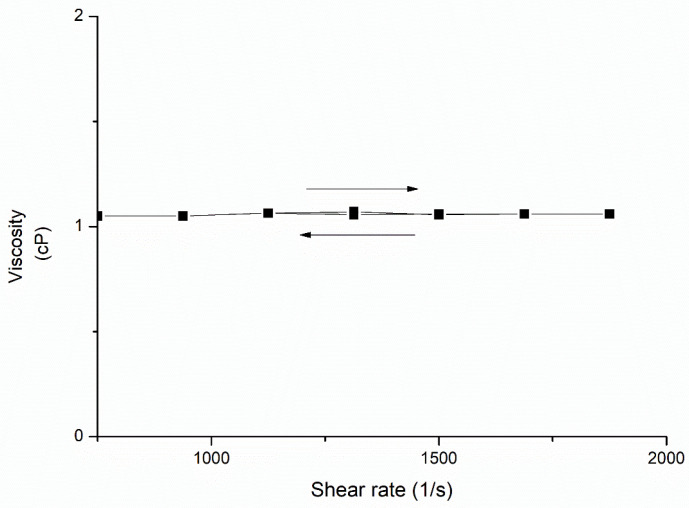
Flow properties of donepezil–NLC dispersion at 25 °C; n = 3.

Rheological analyses were performed for donepezil–NLC dispersions at 25 °C, and the results are shown in Figure 7. Donepezil–NLC dispersions displayed rheological behavior consistent with the Newtonian flow (η = 1.02), characterized by no change in apparent viscosity with increasing shear rates.

### 3.3. In Vitro Drug Release

**Figure 8:**
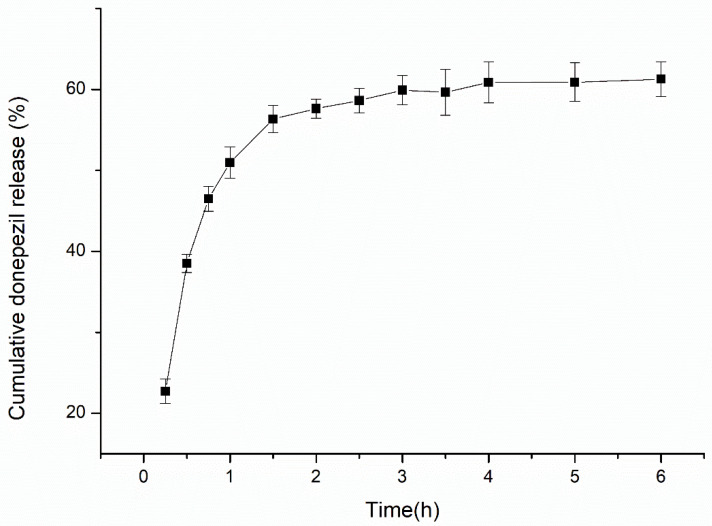
In vitro release profile of donepezil from donepezil–NLC (n = 6).

In vitro drug release studies were performed with donepezil–NLCs to assess the potential of NLCs to control the release of donepezil. The release profile of donepezil from donepezil–NLC is shown in Figure 8, demonstrating an initial burst effect around 30 min followed by slower drug diffusion from the NLCs. Donepezil–NLCs released approximately 25%, 38%, and 56% of the drug at 0.5, 1, and 1.5 h, respectively. The release kinetic model that best fitted the release data was evaluated using the correlation coefficient (r). The kinetic modeling of drug release was performed for 1.5 h, after which a plateau was achieved. The release data from donepezil–NLC followed the Korsmeyer–Peppas model (r = 0.9709 ± 0.0055), indicating non-Fickian diffusion due to the *n*-value of 0.5105 ± 0.0246.

### 3.4. Preliminary Stability Studies

Donepezil–NLC dispersions showed small variations in hydrodynamic diameter, PdI, and zeta potential values up to six months of refrigerated storage (Figure S1). And the EE (%) values remained high (97.680 ± 0.008).

### 3.5. Plasma and Brain Pharmacokinetics

**Figure 9:**
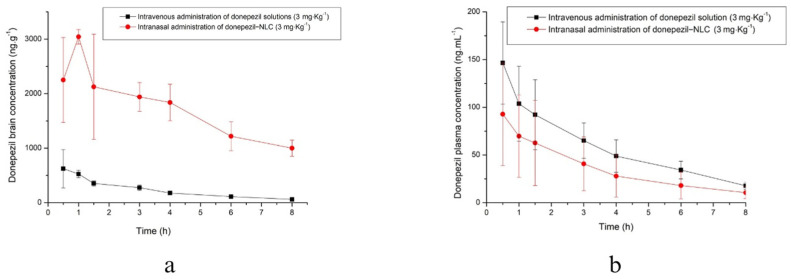
Donepezil concentrations in the brain (**a**) and plasma (**b**) after a single dose of donepezil from the intravenous administration of donepezil solution (3 mg·kg^−1^) and intranasal administration of donepezil–NLC (3 mg·kg^−1^) (n = 6).

Figure 9 illustrates the brain and plasma concentration–time profiles of donepezil in rats after intranasally administering a single dose of donepezil (3 mg·kg^−1^) as donepezil–NLC, as well as after the intravenous administration of donepezil solution (3 mg.kg^−1^). The estimation of the pharmacokinetic parameters in the brain and plasma is shown in Table 2 and Table 3, respectively. These values were derived using a non-compartmental model.

Brain targeting indices were evaluated compared to the intranasal administration of donepezil–NLCs, reaching significant values of DTE (2725.01%) and DTP (96.33%). This suggests a preferential direct nose-to-brain pathway for donepezil to reach the brain following intranasal administration.

## 4. Discussion

The formulation of NLCs depends on using appropriate components for their formulation. Lipids must be selected to increase DL in the system. Therefore, lipids such as stearic acid and oleic acid are components of a solid–liquid binary lipid matrix, which can encapsulate high concentrations of donepezil (98.033% ± 0.0010 EE and 19.2% ± 0.170 DL) due to the high solubility of donepezil in the matrix [48]. Tween 80^®^ was used not only to stabilize the nanoparticles but also for its ability to interact with and inhibit P-glycoprotein, helping to open the junctions between nasal epithelial cells and increase drug permeability [47]. Pluronic F68^®^ provides steric stability to the colloidal dispersion, reduces the viscosity and elasticity of mucus, and improves donepezil diffusion [49]. Additionally, histological studies have shown that Tween 80^®^ and Pluronic F68^®^ are safe surfactants to be used in formulations for nasal administration [25,37]. Therefore, these donepezil–NLC formulations were developed using non-toxic materials, resulting in safer and more stable biodegradable and biocompatible systems. To ensure that the formulations did not irritate the nasal cavity, the donepezil–NLC dispersions were prepared to have a pH compatible with the nasal mucosa (5.5–6.5) and an osmotic pressure of 280 mOsmol·Kg^−1^. This is an important feature considering that irritating the nasal mucosa may change drug permeability [8,20].

The characterization of nanocarriers requires the determination of their size and distribution because they alter drug disposition, such as absorption, biodistribution, and the route of elimination. Particles with a diameter of up to 100 nm have been reported to be more effective for nose-to-brain targeting [45,47,50]. The DLS measurements of donepezil–NLC showed nanometric and homogeneous size (Table 1), which is considered promising for brain targeting via the nasal route. AFM images showed particles with a spherical shape, regular surface, and smaller size than those observed in the DLS measurements (Figure 1). This difference in size measurement was attributed to the analytical techniques used since the DLS technique measures the hydrodynamic diameter when nanocarriers are swollen, while AFM measures nanocarriers in their dry state. Furthermore, AFM images showed that drug incorporation caused a decrease in particle size. This is likely related to the lipophilic characteristics of donepezil, which make the lipid matrix more compact due to intermolecular interactions between the drug and the NLC lipids, leading to the retraction of the particles and a decrease in particle size [27,51,52].

Another important parameter to be analyzed in the development of nanocarriers is the physical stability of these systems during storage. This property is related to the constant collisions among nanocarriers as they move according to the diffusive forces caused by Brownian motion [50]. To avoid the flocculation or agglomeration of the nanocarriers, some polymers (e.g., Pluronic F68^®^) or nonionic surfactants (e.g., Tween 80^®^) may be adsorbed on the surface of the nanocarriers to stabilize the colloidal dispersion by a steric mechanism [8,49]. Moreover, nanocarriers in aqueous dispersions can be stabilized by the presence of a higher surface charge, in modulus, ≥30 mV, promoting electrostatic repulsion among these nanoparticles, thus hindering the flocculation and agglomeration process [12,50]. Zeta potential values greater than ׀30׀ mV were achieved for the NLCs (Table 1), indicating the physical stability of these colloidal dispersions. Preliminary studies also indicated the physical stability of donepezil–NLC dispersions for up to 180 d of storage under refrigeration (Figure S1). Indeed, large molecules such as Tween 80^®^ and Pluronic F68^®^ have been shown to maintain long-term physical stability through steric stabilization [8,12,49].

The physical stability of lipid nanoparticles is also related to the solid nature and crystallinity of carrier storage [46,47]. Furthermore, the crystallinity of NLCs can influence drug encapsulation and release [53]. DSC studies demonstrated the crystalline nature of the main components of the formulation and the absence of endothermic events related to the melting point of stearic acid and donepezil in the DSC curves of loaded NLCs (Figure 2), suggesting the formation of an amorphous lipid matrix and drug solubilization in the lipid matrix [46,47]. The crystallinity of the NLCs was also assessed by PXRD (Figure 3), and the diffractogram of donepezil–NLC did not show the peaks of the drug, suggesting that donepezil loaded in the crystal lattice of lipids is amorphously or molecularly dispersed within the lipid matrix [48,52]. Analyses of the physical mixture of stearic acid, donepezil, and mannitol confirmed the absence of donepezil crystals in the donepezil–NLC diffraction pattern due to the incorporation of donepezil into the lipid matrix. The comparative PXRD pattern (Figure 4) confirmed that the drug was entrapped in the lipid matrix, which was desirable when the drug was loaded into nanocarriers. Together, the DSC and PXRD studies demonstrated that drug solubilization within the lipid matrix of NLCs in an amorphous form potentially provides better stability during storage and modified drug release properties and can modify drug disposition in the body [46,47].

The characterization of NLCs by FTIR was performed to investigate the structural properties of lipids and the interactions between drugs and other components of NLCs [54,55]. FTIR analyses showed the main absorption bands of the formulation components and the lack of bands characteristic of donepezil in the NLC spectra (Figure 5). These findings suggested the incorporation of the drug into the lipid matrix of the nanocarrier [52]. Additionally, we observed the characteristic bands of donepezil in the spectra of the physical mixtures of stearic acid, donepezil, and mannitol (Figure 6), indicating that the analyses could identify the drug in the presence of other components of the sample. Therefore, we confirmed that donepezil was incorporated into the lipid matrix of NLCs.

Rheological studies can help characterize the flow properties of NLC dispersions. It was shown that dispersions of lipid nanocarriers have elastic properties only when nanocarriers are dispersed at high concentrations. In this case, a transition from a low-viscosity liquid dispersion to an elastic system with a continuous network of lipid nanocarriers may be observed [56,57]. However, the donepezil–NLCs showed Newtonian flow (Figure 7), which may be related to the low concentration of lipids in the sample. Diluted dispersions are a feature of NLCs prepared using the hot microemulsion technique [38]. Thus, the system shows low viscosity, an interesting feature from a pharmaceutical point of view, enabling easy application and good spreadability into the nasal cavity.

Release studies are important for predicting the performance of novel formulations in vivo. Once NLCs are loaded with a drug that is soluble in the lipid matrix, prolonged drug release properties may be achieved. One of the main characteristics of the nasal route is the short time that the formulation remains in the nasal cavity [49]. Thus, it is expected that the nasal formulation will release the drug within a short period. Considering the possibility of nanocarriers targeting donepezil to the brain via the olfactory route [13], sustained release would be interesting. The release profile of donepezil from NLCs demonstrated an initial burst (releasing around 25% in 30 min) followed by sustained release (38% and 56%, respectively, at 1.0 and 1.5 h) (Figure 8). This biphasic drug release pattern from NLCs has been observed by others [27,48,54,58,59,60], and the burst effect and sustained release were related to the drug incorporated on the surface of the NLC and into its lipid matrix. Particularly in hot preparation methods, during the rapid cooling of lipids, drug partitioning from the liquid lipid phase to the aqueous phase favors the formation of a drug-rich wall particle and leads to an initial burst release [27,48,54,61]. The sustained release pattern is attributed to the strong interactions of the drug with the lipid matrix due to its high lipophilicity and the slow degradation of NLCs in the release medium [48]. A large amount of donepezil was released throughout 1.5 h, which was attributed to non-Fickian drug diffusion from the lipid matrix, possibly associated with some degree of swelling and erosion, as suggested by the *n*-value found in the Korsmeyer–Peppas kinetic model (*n*-value > 0.5). The evidence of anomalous diffusion was reinforced by analyzing the minor adjustment to the Higuchi model (0.9491 ± 0.0079) compared to Korsmeyer–Peppas (0.9709 ± 0.0055), considering that the Higuchi model better fits drug release profiles governed by Fickian diffusion.

The effect of the intranasal administration of donepezil–NLC on brain and plasma pharmacokinetic parameters is highlighted when compared to the intravenous administration of a donepezil solution (Figure 9). The intranasal administration of donepezil–NLC showed a C_max_ value in the brain 4.7 times higher than that of the intravenous solution, and the AUC^0–ꝏ^ value in the brain was 10.5 times higher than that of the intravenous solution (Table 2). Considering that the intravenous administration of a drug dose is fully bioavailable (absolute bioavailability), these results were attributed to nose-to-brain targeting via the olfactory and trigeminal neural pathways. The nasal route enables the direct transport of drugs to the brain, and reduced systemic distribution in the blood may be achieved, probably reducing the common systemic side effects of the drug [29,30,37,47]. In a previous study, we evaluated the brain and plasma pharmacokinetics of intranasal and oral donepezil solutions and one nasal formulation (based on a liquid crystal). The findings showed that nasal delivery resulted in the rapid absorption of donepezil in the brain (with T_max_ decreasing) compared with oral administration [40]. In this study, donepezil–NLC delivered via the nasal route demonstrated a T_max_ in the brain (Table 2), consistent with our earlier results. Furthermore, the higher brain T_max_ for intranasal donepezil–NLCs compared to intravenous donepezil solutions may be attributed to the sustained release of donepezil from NLCs, which is strongly related to drug targeting to the brain by direct transport. Considering that donepezil is an acetylcholinesterase inhibitor, the pharmacodynamics of novel drug delivery systems targeting donepezil in the brain may enable improved efficacy in treating Alzheimer’s disease and, at the same time, improve safety when avoiding the peripheral inhibition of acetylcholinesterase. This is supported by plasma parameters, showing that the C_max_ and AUC^0–ꝏ^ of the intravenous solution were 2.3 and 2.6 times higher than those of intranasal donepezil–NLC (Table 3), corroborating our hypothesis that direct transport was reached, with drug targeting to the brain. T_max_ values, however, were similar for both groups, indicating fast absorption through the nasal route (Table 3 and Figure 9). This might be due to the lipid nature of the developed system, the nano-sized particles, and the previously discussed effect of Tween 80^®^ and Pluronic F68^®^ in increasing drug permeability. Therefore, we can infer that a fraction of the drug was quickly absorbed by the systemic route, while another portion was directed to the brain, resulting in increased concentrations and bioavailability in this region. The percentage of drugs that reached the brain directly was also calculated using intravenous parameters. Following the intranasal delivery of donepezil–NLCs, high levels of % DTE (2725.01) and % DTP (96.33%) were observed, suggesting a preferential direct nose-to-brain route for donepezil to reach the brain. It is important to note that DTE values may be influenced not only by increased brain exposure but also by reduced systemic (plasma) concentrations. Therefore, DTE should be interpreted together with DTP values and absolute pharmacokinetic profiles to avoid the overestimation of brain targeting efficiency. These preferential direct nose-to-brain pathways may reduce the systemic availability of donepezil compared to the IV route, resulting in less toxicity for the main peripheral organs of patients who need long-term drug administration.

After intranasal administration, drug transport to the brain may occur through olfactory and trigeminal neural pathways, allowing for direct access to the central nervous system and bypassing the blood–brain barrier. This transport is influenced by the physicochemical properties of the nanocarrier system, such as particle size and lipid composition, which may facilitate interaction with the nasal epithelium and neuronal pathways.

Donepezil exerts its pharmacological effect by inhibiting acetylcholinesterase, leading to increased acetylcholine levels in the brain and improvement in cognitive function. Although region-specific brain distribution was not evaluated in this study, the increased brain exposure observed suggests effective delivery to central compartments relevant for its pharmacological action.

One limitation of this study is the absence of an intranasal donepezil solution as a control group. Although such a comparison could provide additional insight into the contribution of the nanocarrier system, previous studies from our group have already demonstrated the pharmacokinetic profile of intranasal donepezil solutions [40]. Therefore, the present study was designed to evaluate whether the nanostructured lipid carrier system could further enhance brain targeting compared to systemic administration.

Another limitation is that brain samples were collected without prior saline perfusion, and therefore a residual contribution from blood in cerebral capillaries cannot be completely excluded. In addition, although terminal sampling was adopted to enable the direct quantification of donepezil in brain tissue, alternative approaches such as CSF sampling may be considered in future studies to reduce animal use. However, CSF concentrations do not necessarily reflect drug distribution in brain tissue, particularly in studies involving nanocarrier systems.

## 5. Conclusions

This study describes the obtainment of donepezil-loaded NLCs for intranasal administration and direct nose-to-brain targeting. The physical stability, high drug entrapment, and biocompatibility with the nasal cavity aid in our aim to obtain donepezil–NLCs. The drug was also well absorbed and exhibited a suitable release profile. Compared to the intravenous administration of donepezil solution, the intranasal administration of donepezil–NLCs significantly increased brain bioavailability, and drug concentrations were sustained, suggesting a preferential direct nose-to-brain pathway for donepezil based on the pharmacokinetics of donepezil in the brains of rats. Therefore, donepezil–NLC was developed as an efficient drug delivery system for the nose to the brain, which may result in improved Alzheimer’s disease therapy in the future by allowing for the use of lower doses of donepezil with fewer side effects. Furthermore, donepezil–NLC formulations can be safe due to the nature of their components and the fact that they minimize the toxicity of the encapsulated drug. However, cytotoxicity studies should be conducted preliminarily as indicators of the safety of donepezil–NLC.

## Figures and Tables

**Table 1:** Hydrodynamic diameter, PdI, zeta potential, EE, and DL for NLC dispersions.

Samples	Hydrodynamic Diameter (nm)	PdI	Zeta Potential (mV)	EE (%)	DL (%)
Unloaded NLCs	111.37 ± 4.500	0.280 ± 0.044	−42.23 ± 0.473	---	---
Donepezil–NLC	110.03 ± 1.55	0.262 ± 0.024	−39.80 ± 0.251	98.033 ± 0.001	19.2 ± 0.170

Polydispersity index (PdI), entrapment efficiency (EE), and drug loading (DL). The results represent the average (±standard deviation) of three determinations.

**Table 2:** Pharmacokinetic parameters in the brain after a single dose of donepezil from the intravenous administration of donepezil solution (3 mg·kg^−1^) and the intranasal administration of donepezil–NLC (3 mg·kg^−1^) (n = 6).

Pharmacokinetic Parameters	Intravenous Donepezil Solution (3 mg·kg^−1^)	Intranasal Donepezil–NLC (3 mg·kg^−1^)
AUC^0–ꝏ^ (h.ng/g)	1922 (1605–2693)	20,206 (15,412–24,752)
T_max_ (h)	0.50 (0.50–1)	1 (1–1.5)
C_max_ (ng·g^−1^)	667.3 (515.3–958.6)	3117 (2846–3976)
t_1/2_ (h)	2.085 (1.88–4,19)	4.54 (3.87–5.61)
Clearance (mL/h/kg)	0.0016 (0.011–0.0019)	0.00015 (0.00012–0.00019)

Median (confidence interval). Mann–Whitney nonparametric *t*-test.

**Table 3:** Pharmacokinetic parameters in plasma after single dose of donepezil from intravenous administration of donepezil solution (3 mg·kg^−1^) and intranasal administration of donepezil–NLC (3 mg·Kg^−1^) (n = 6).

Pharmacokinetic Parameters	Intravenous Donepezil Solution (3 mg·kg^−1^)	Intranasal Donepezil–NLC (3 mg·kg^−1^)
AUC^0–ꝏ^ (h·ng/mL)	585.8 (379.0–806.9)	226.0 (108.5–313.6)
T_max_ (h)	0.5 (0.5–0.5)	0.5 (0.5–0.5)
C_max_ (ng·mL^−1^)	143.3 (91.98–197.3)	61.39 (52.16–101.4)
t_1/2_ (h)	3.04 (2.48–3.29)	2.64 (1.45–3.19)
Clearance (mL/h/kg)	0.0055 (0.0040–0.0080)	0.014 (0.010–0.028)

Median (confidence interval). Mann–Whitney nonparametric *t*-test.

## Data Availability

The raw data supporting the conclusions of this article will be made available by the authors on request.

## Supplementary Materials

The following supporting information can be downloaded at https://www.mdpi.com/article/10.3390/pharmaceutics18050541/s1, Figure S1: Physical stability of donepezil–NLC dispersions evaluated by hydrodynamic diameter, PdI, and zeta potential measurements.
