# Supplementary material for: Nanostructured Lipid Carriers Loaded with Donepezil for Nose-to-Brain Targeting
Source: Pharmaceutics. 2026 Apr 28;18(5):541. doi: 10.3390/pharmaceutics18050541 (PMC13211091; doi:10.3390/pharmaceutics18050541)
Supplement: Supplementary file 1 [file pharmaceutics-18-00541-s001.zip › pharmaceutics-4204314-supplementary.pdf]

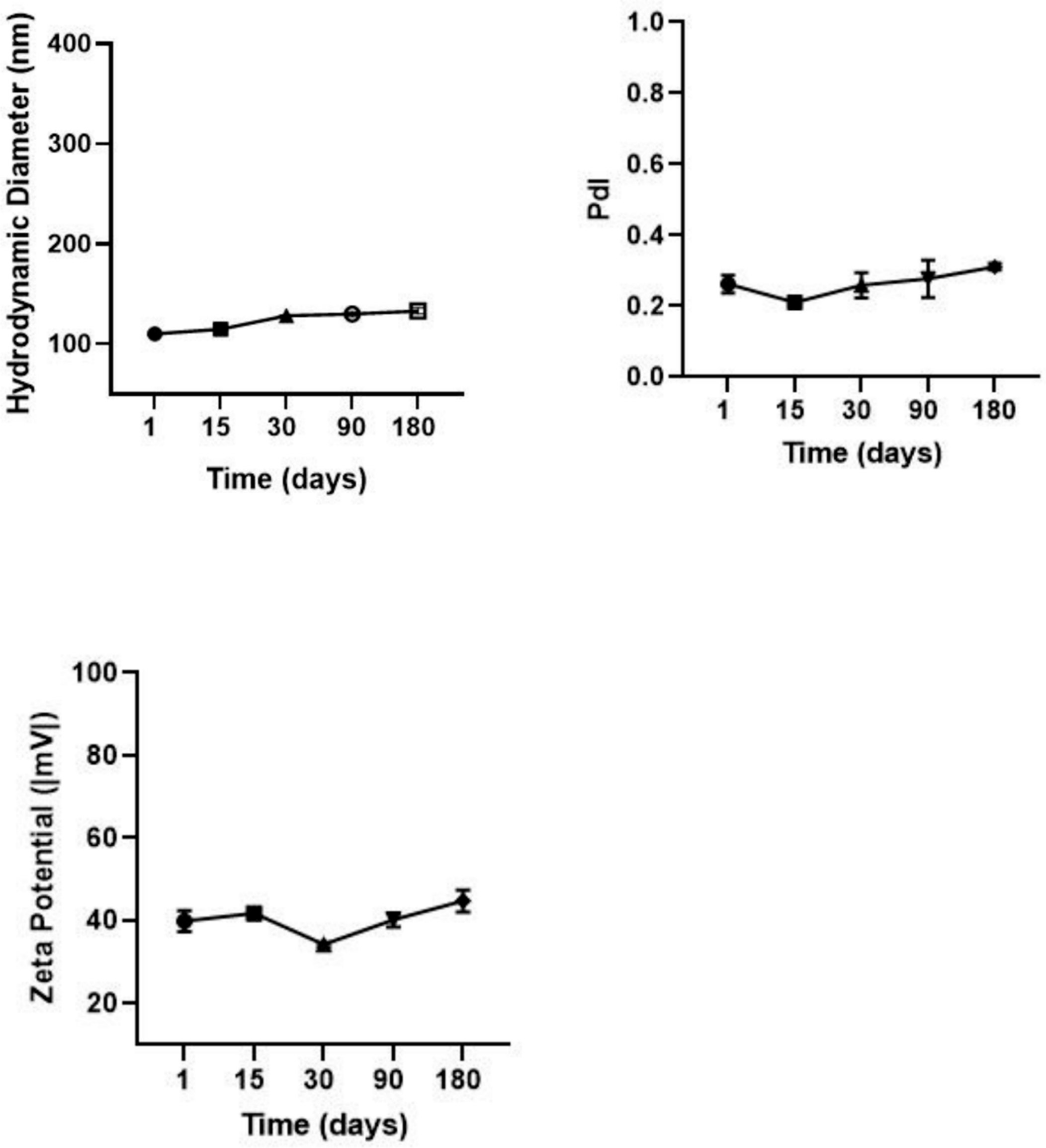

Figure S1. Physical stability of donepezil-NLC dispersions evaluated by hydrodynamic diameter, PdI, and zeta potential measurements.
